# Age-Specific Differences in Online Grocery Shopping Behaviors and Attitudes among Adults with Low Income in the United States in 2021

**DOI:** 10.3390/nu14204427

**Published:** 2022-10-21

**Authors:** Pasquale E. Rummo, Christina A. Roberto, Lorna E. Thorpe, Andrea B. Troxel, Brian Elbel

**Affiliations:** 1 Department of Population Health, New York University Grossman School of Medicine, 180 Madison Avenue, 3rd Floor, New York, NY 10016, USA; 2 Perelman School of Medicine, University of Pennsylvania, Philadelphia, PA 19104, USA; 3 Wagner Graduate School of Public Service, New York University, New York, NY 10012, USA

**Keywords:** food security, older adults, internet, disparities

## Abstract

Background: Online grocery shopping has surged in popularity, but we know little about online grocery shopping behaviors and attitudes of adults with low income, including differences by age. Methods: From October to November 2021, we used a survey research firm to recruit a convenience sample of adults who have ever received Supplemental Nutrition Assistance Program (SNAP) benefits (*n* = 3526). Participants completed an online survey designed to assess diet and online food shopping behaviors. Using logistic regression, we examined the relationship between participant characteristics, including age, and the likelihood of online grocery shopping, and separately examined variation in the reasons for online grocery shopping by age. Results: About 54% of the participants reported shopping online for groceries in the previous 12 months. Odds of online shopping were higher for those aged 18–33 years (OR = 1.95 (95% CI: 1.52, 2.52; *p* < 0.001)) and 34–44 years (OR = 1.50 (95% CI: 1.19, 1.90; *p* < 0.001)) than for those aged ≥65 years. Odds were also higher for those who were food insecure and those with income below USD 20,000, higher educational attainment, and higher fruit and vegetable intake. Low prices were the most popular reason for online grocery shopping (57%). Adults aged 18–33 years old had higher odds of reporting low prices as a motivating factor than older adults (OR = 2.34 (95% CI: 1.78, 3.08; *p* < 0.001)) and lower odds of reporting being discouraged by lack of social interaction (OR = 0.34 (95% CI: 0.25, 0.45; *p* < 0.001)). Conclusion: Strategies for making online grocery shopping more affordable for adults with lower income may be promising, especially online produce. For older adults, additional support may be needed to make online shopping a suitable replacement for in-store shopping, such as education on technology and combining it with opportunities for social support.

## 1. Introduction

To reduce food insecurity, the United States Department of Agriculture (USDA) offers financial assistance through the Supplemental Nutrition Assistance Program (SNAP) program, delivering nutrition benefits to over 41 million households [1]. Between 2019 and 2021, food insecurity grew by over 15% in the U.S., largely due to a surge in unemployment and income loss during the COVID-19 pandemic [1]. During this period, online grocery shopping rapidly expanded in popularity, and now accounts for 10% of all U.S. grocery sales [2]. Online food shopping may promote healthy choices by mitigating the influence of in-store triggers and support equitable food access [3,4], but it may also lead to more frequent purchases of less healthy foods due to targeted marketing [5].

In response to increased demand for online grocery shopping options, the USDA expanded the SNAP Online Purchasing Pilot (OPP) program to additional retailers and locations [6]. The program allows SNAP recipients to use their benefits in online transactions [7]. Partly fueled by the pandemic shutdown, the value of SNAP benefits redeemed online grew from USD 2.9 million in February 2020 to USD 196.3 million in September 2020, reaching 2.4% of total SNAP sales [8]. Research shows, however, that online grocery delivery services are inequitably distributed for those paying with SNAP benefits, with lower access in rural areas and areas with higher poverty and limited food access [9,10,11].

A recent review study highlighted several reasons for the low uptake of online grocery shopping among those with low income, including high cost and lack of control over food selection, lack of social interaction, and lack of interest [12]. Several benefits were also reported, such as lower stress, saving time, and fewer impulse purchases than in-store shopping. Another study found that purchases of fresh fruits and vegetables were lower online than in-store in SNAP-eligible households [13]. The majority of this previous work has focused on small geographic areas, with small samples, and thus lacked sufficient statistical power to test for differences by respondent characteristics. A more recent study, however, used a large, nationally representative sample of mostly food-secure adults and found that 39% had ever shopped online for groceries [14].

In this study, we characterized online grocery shopping behaviors and attitudes in a nationwide sample of adults with low income. We examined the extent to which the frequency of online grocery shopping differed by age and other sociodemographic characteristics and the frequency of fruit and vegetable intake. Given that younger (vs. older) individuals are more likely to use the internet and shop online [12], we also examined whether other behaviors and attitudes related to online grocery shopping differed by age.

## 2. Materials and Methods

### 2.1. Data

This study was part of a grant designed to characterize online grocery shopping behaviors and attitudes in adults with low income and examine the extent to which financial incentives and behavioral nudges increased fruit and vegetable purchases in a randomized, controlled experiment. To achieve the goals of our grant, we used CloudResearch, a survey research firm, to recruit a convenience sample of adults aged 18 years or older who have ever received SNAP benefits, read and speak English, and live with fewer than four people (to accommodate the shopping budget for the randomized, controlled experiment of the parent grant). The sample was recruited to approximately match the distribution of gender and age of adults residing in the U.S. in 2019 [15], using non-random quota sampling from participant pools on several market research platforms. Invitations were sent to eligible participants via email and dashboards. CloudResearch has quality control mechanisms, such as English comprehension screener questions and attention checks, and those participants who complete CloudResearch’s online surveys receive points that can be redeemed for various incentives, including cash, lotteries, or donations to charity.

The survey was completed on a personal computer, laptop, tablet, or mobile phone from October to November 2021. Qualtrics, an online survey platform, was used to create and distribute the survey (Supplemental File S1) [16]. The survey was designed to assess sociodemographic characteristics, health status, food shopping behaviors, and fruit and vegetable intake. Sociodemographic and food insecurity questions were derived from the 2017–2018 National Health and Nutrition Examination Survey [17]. The questions related to grocery shopping were derived from the USDA National Household Food Acquisition and Purchase Survey (FoodAPS) [18] or adapted from previous work by the authors [19,20]. We captured weekly fruit and vegetable intake using a 6-item fruit and vegetable dietary intake module from the Behavioral Risk Factor Surveillance System [21].

### 2.2. Outcomes

Our primary outcome was whether the participants reported ever shopping online for groceries in the previous 12 months. Among those who shopped online for groceries, we estimated the frequency of online grocery shopping, types of groceries purchased online, types of retailers, and methods of delivery. We also calculated the frequency (%) of reasons that the participants choose as motivating or preventing them from purchasing groceries online.

### 2.3. Statistical Analysis

To determine the extent to which our convenience sample differs from a national probability sample, we used binomial tests and *t*-tests to compare the sociodemographic characteristics of our sample to respondents in the FoodAPS survey who ever participated in SNAP. We report descriptive results using averages and standard deviations, or median and interquartile range. Using logistic regression, we examined the relationship between our primary outcome and sociodemographic characteristics, including age group (quartiles: 18–33, 34–44, 45–59, ≥60 years), gender, household size (total and children), ethnicity (yes/no Hispanic or Latinx), race (White; Black; Asian, Native Hawaiian, or Pacific Islander; American Indian or Alaska Native; and Other), educational attainment (yes/no high school or less), household income (yes/no <USD 20,000 annually), marital status (yes/no married), employment status (yes/no currently employed or student), SNAP status (yes/no currently receiving benefits), responsible for most household food shopping (yes/no), and responsible for most household food preparation (yes/no). The model also included food insecurity status, defined as yes if a participant indicated that it was true or sometimes true that (1) their household was worried whether their food would run out before they had money to buy more, and/or (2) the food that they bought did not last and they did not have enough money to buy more. We also included fruit and vegetable intake, which we transformed into times per week using the median of response options, and then categorizing responses into quartiles. For age group, race, and fruit and vegetable intake, we performed likelihood ratio tests to determine whether the odds of online grocery shopping differed among the levels overall; we then performed pairwise comparisons if the results were statistically significant. In additional analyses, we used logistic regression to examine the relationship between other online grocery shopping behaviors and attitudes and age group, controlling for other covariates. We used a two-sided alpha of 0.05 as the threshold for statistical significance in our primary analysis. In our additional analyses (*n* = 60), we used a *p* < 0.0008 significance threshold (0.05/60) and corrected for multiple comparisons using the Bonferroni–Holm procedure [22].

## 3. Results

We excluded those participants who reused the same IP address (*n* = 265) and/or did not finish the survey (*n* = 90). We also excluded those who finished the survey in under one-third of the median completion time (<2.1 min) (*n* = 51). The final sample included 3526 adults, and the median completion time for the survey was 11.9 min. Approximately 51% of the sample identified as female, and the average age was 46.8 (SD = 15.9) years (Table 1). The average household size was 2.3 (SD = 1.0), including 1.4 (SD = 0.7) children per household. The majority of the sample identified as non-Hispanic/Latinx (90.0%) and/or White (75.2%). About 44% of the participants reported an annual household income <USD 20,000, 58.6% reported being unemployed, 67.0% reported currently receiving SNAP benefits, and 70.3% were classified as food insecure. On average, the participants reported consuming fruits and vegetables 16.7 (SD = 16.8) times per week. Compared with the FoodAPS sample, our sample had a higher percentage of older participants and participants who identified as White and non-Hispanic/Latinx, and a higher percentage of participants with household income <USD 20,000.

In the full sample, 54% reported shopping online for groceries in the previous 12 months, primarily via Walmart (38%) or Amazon (19%) (Table 2). The likelihood ratio tests indicated that the odds of online grocery shopping differed across levels of age group, race, and fruit and vegetable intake (*p* values < 0.001). The model-based results indicate that the odds of online grocery shopping were higher for those aged 18–33 years (OR = 1.95 (95% CI: 1.52, 2.52)) and 34–44 years (OR = 1.50 (95% CI: 1.19, 1.90)) than for those 65 years or older, and higher for households with more children (OR = 1.24 for every additional child (95% CI: 1.07, 1.43)) (Table 3). Those who identified as Hispanic/Latinx (OR = 1.63 (95% CI: 1.21, 2.19)) or Black (OR = 1.52 (1.22, 1.89)) had higher odds of online grocery shopping than non-Hispanic/Latinx and White participants, respectively. The odds of online grocery shopping were lower for those with a high school education or less (OR = 0.83 (95% CI: 0.71, 0.97)) and income <USD 20,000 per year (OR = 0.81 (95% CI: 0.68, 0.96)), and higher for those who were employed (OR = 1.43 (95% CI: 1.20, 1.69)) or food insecure (OR = 1.42 (95% CI: 1.20, 1.67)). The odds of online grocery shopping were also higher for those with a higher self-reported intake of fruits and vegetables.

Among those who shopped online for groceries, 54% reported shopping online at least once a month, and 18% at least once per week (Table 2). Fresh produce (19%) and desserts, snacks, and candy (18%) were the most popular items purchased online, whereas meat, poultry, and fish (12%), and grains (11%) were less popular. About 57% reported low prices as a motivating factor, and 32–40% of the participants reported being motivated by a good selection of produce, good quality food, the variety of goods, having someone else select grocery items on their behalf, and/or inexpensive or no delivery fees. Adults aged 18–33 years old had higher odds of reporting low prices as a motivating factor than older adults (OR = 2.34 (95% CI: 1.78, 3.08; *p* < 0.001)), with an 11% difference between age groups (Table 4). We observed similar associations between age group and other motivating reasons, including the variety of goods, good quality food, having someone else select grocery items on one’s behalf, and having an option for using SNAP benefits online. 

Among those who reported never shopping online for groceries, 64% reported a lack of social interaction as a reason preventing them from shopping online, and 27–29% of the participants reported high prices, not being able to interact with the food itself, and/or a lack of a loyalty/frequent shopping program to be the reasons preventing them from shopping online (Table 2). Adults aged 18–33 years old had lower odds of reporting being discouraged by lack of social interaction than older adults (OR = 0.34 (95% CI: 0.25, 0.45; *p* < 0.001)) (Table 4). Only 5% of the participants reported that the lack of an option for using SNAP benefits online was a discouraging factor, with no significant differences by age group.

## 4. Discussion

We found that a little over half of the participants with lower income reported shopping online for groceries at least once in the previous 12 months, which is higher than the frequency estimates published prior to the pandemic [14,19,23] but similar to more recent estimates collected during the pandemic [13], which might reflect a combination of a surge in online shopping during the lockdown and the expansion of the SNAP OPP program in 2020–2021. A third of the sample reported that the lack of social interaction discouraged them from ever shopping online, which is consistent with previous work [12] and also suggests that for some, especially older adults, online grocery shopping is not a suitable replacement for in-store shopping.

Though some fresh items, such as meat, poultry, and fish, were less frequently purchased online, almost a fifth of our sample reported shopping online for fresh produce, and a good selection of produce was a popular motivation to shop online, in addition to low prices. Indeed, those who consumed more fruits and vegetables per week were more likely to report shopping online for groceries. This is lower than reported in a recent study, which found that about half of mostly food-secure adults purchased fresh foods [14]. Taken together, these findings suggest that financial incentive programs targeting fruits and vegetables may be attractive and effective options to promote healthy purchasing behaviors among SNAP participants shopping in online retail settings. This may be an especially effective option for older adults, who had a lower percentage of purchasing fresh produce online relative to younger adults.

Like previous studies [12], we found that younger individuals were more likely to shop online than their older counterparts, seemingly driven by lower prices and convenience, including the option to use SNAP benefits online. These results suggest that further expansion of the SNAP OPP program may be an effective strategy for motivating younger adults to start shopping online for groceries but may not be sufficient to motivate older adults with low income. Older adults were particularly discouraged by the lack of social interaction and may instead benefit from an expansion of “click-and-collect” options, wherein customers order groceries online for pick-up at a centralized location, such as a community center; or the delivery of groceries with a social support component (e.g., checking in to see how the customer is doing and providing additional resources as needed).

We also found those with higher food insecurity were more likely to shop online, which is consistent with another study that found a particularly high prevalence of online grocery shopping among higher-income food-insecure households, potentially due to job loss during the pandemic or limited food access [14]. Unlike that study, however, we found that participants who identified as Black and Hispanic/Latinx had higher odds of shopping online for groceries. This may be because Black and Hispanic/Latinx individuals are more likely to live in urban areas where online grocery shopping options may be more prevalent or where there is limited access to neighborhood supermarkets. It may also reflect differences in norms, preferences, and attitudes across racial/ethnic groups.

Though our sample was recruited to match the distribution of gender and age of adults in the U.S., our sample was not nationally representative of SNAP-participating adults. Yet, it was larger and more geographically diverse than the samples in previous studies, which allowed us to examine unique relationships between participant characteristics and online grocery shopping frequency. Another limitation of our study is that the distribution of sociodemographic characteristics in our sample differed in some ways from that of the FoodAPS sample, and it was not recruited using random sampling. However, previous studies indicate that experimental results from convenience samples can yield similar findings to the results of studies conducted via probability-based samples, despite differences in demographic characteristics between samples [24,25,26]. Overall, our findings highlight the need to develop and test strategies for making online grocery shopping more affordable and appealing for individuals with lower income. Future research should strive to understand why specific groups are more likely to shop online for groceries, such as those in food-insecure households, and the extent to which their purchases differ from their counterparts who primarily shop in-store.

## Figures and Tables

**Table 1:** Sociodemographic characteristics and diet behaviors ^a^.

	Mean (SD) or *n* (%)
Age	46.8 (15.9)
Female	1808 (51.3%)
Household size, total	2.3 (1.0)
Household size, children <18 years	1.4 (0.7)
Hispanic, Latino, or Spanish	352 (10.0%)
Race	
American Indian or Alaska Native	51 (1.4%)
Asian	52 (1.5%)
Black or African American	523 (14.8%)
Native Hawaiian or Pacific Islander	12 (0.3%)
White	2652 (75.2%)
Other	90 (2.6%)
More than 1	17 (0.5%)
Prefer not to answer	129 (3.7%)
Education	
Less than 9th grade	35 (1.0%)
9th to 12th grade–No diploma	206 (5.8%)
High school graduate	1004 (28.5%)
GED or equivalent	235 (6.7%)
Some college, no degree	1077 (30.5%)
Associate’s degree	439 (12.5%)
Bachelor’s degree	386 (10.9%)
Graduate or professional degree	140 (4.0%)
Prefer not to answer	4 (0.1%)
Income	
<USD 20,000	1546 (43.8%)
USD 20,000–39,999	1295 (36.7%)
USD 40,000–59,999	435 (12.3%)
USD 60,000–USD 79,999	138 (3.9%)
USD 80,000–USD 99,999	47 (1.3%)
USD 100,000–119,999	9 (0.3%)
USD 120,000 to USD 139,999	3 (0.1%)
USD 140,000 to USD 159,999	8 (0.2%)
USD 160,000 to USD 179,999	3 (0.1%)
USD 180,000 to USD 199,999	3 (0.1%)
≥USD 200,000	8 (0.2%)
Don’t know	16 (0.5%)
Prefer not to answer	15 (0.4%)
Marital status	
Married	770 (21.8%)
Widowed	236 (6.7%)
Divorced	613 (17.4%)
Separated	154 (4.4%)
Never married	1185 (33.6%)
Living with partner	550 (15.6%)
Prefer not to answer	18 (0.5%)
Employment	
Working at a job or business	1086 (30.8%)
With a job or business but not at work	94 (2.7%)
Looking for work	631 (17.9%)
Not working at a job or business	1435 (40.7%)
Part-time or full-time student	135 (3.8%)
Prefer not to answer	145 (4.1%)
Food insecure ^b^	2479 (70.3%)
SNAP participation, currently	2364 (67.0%)
BRFSS 2017 screener, times per week	
Fruit juice	2.7 (4.2)
Fruit	3.8 (4.6)
Beans	2.0 (3.1)
Dark green vegetables	2.8 (3.6)
Orange-colored vegetables	2.1 (3.5)
Other vegetables	3.4 (3.9)
Total	16.7 (16.8)
Responsible for most of household food shopping	
Yes	2999 (85.1%)
No	282 (8.0%)
No one person is responsible	245 (6.9%)
Responsible for most of household food preparation	
Yes	2840 (80.5%)
No	453 (12.8%)
No one person is responsible	233 (6.6%)

Note: SNAP = Supplemental Nutrition Assistance Program; BRFFS = Behavioral Risk Factor Surveillance System. ^a^ Sociodemographic and food insecurity questions were derived from the 2017–2018 National Health and Nutrition Examination Survey. We captured weekly fruit and vegetable intake using a 6-item fruit and vegetable dietary intake module from the Behavioral Risk Factor Surveillance System. ^b^ Food insecurity defined as yes if a participant indicated that it was true or sometimes true that (1) their household was worried whether their food would run out before they had money to buy more, and/or (2) the food that they bought just did not last, and they did not have enough money to buy more.

**Table 2:** Grocery shopping behaviors and attitudes: ^a^ overall and by age group ^b^.

	All (*n* = 3526)	18–33 Years (*n* = 857)	34–44 Years (*n* = 892)	45–59 Years (*n* = 889)	60+ Years (*n* = 888)
	*n* (%)	*n* (%)	*n* (%)	*n* (%)	*n* (%)
Online grocery, frequency					
Never	1621 (46.0%)	275 (32.1%)	373 (41.8%)	478 (53.8%)	495 (55.7%)
1 time per month or less	883 (25.0%)	247 (28.8%)	240 (26.9%)	203 (22.8%)	193 (21.7%)
2–3 times per month	684 (19.4%)	214 (25.0%)	188 (21.1%)	145 (16.3%)	137 (15.4%)
1 time per week	217 (6.2%)	73 (8.5%)	55 (6.2%)	45 (5.1%)	44 (5.0%)
More than 1 time per week	121 (3.4%)	48 (5.6%)	36 (4.0%)	18 (2.0%)	19 (2.1%)
Online grocery, retailer ^c^					
Amazon	668 (35.1%)	229 (39.3%)	165 (31.8%)	157 (38.2%)	117 (29.8%)
Walmart	1342 (70.4%)	436 (74.9%)	388 (74.8%)	269 (65.5%)	249 (63.4%)
Target	275 (14.4%)	147 (25.3%)	70 (13.5%)	34 (8.3%)	24 (6.1%)
Costco	143 (7.5%)	69 (11.9%)	32 (6.2%)	25 (6.1%)	17 (4.3%)
Kroger	333 (17.5%)	115 (19.8%)	92 (17.7%)	66 (16.1%)	60 (15.3%)
Whole Foods	147 (7.7%)	69 (11.9%)	40 (7.7%)	19 (4.6%)	19 (4.8%)
Aldi	303 (15.9%)	106 (18.2%)	74 (14.3%)	55 (13.4%)	68 (17.3%)
Publix	175 (9.2%)	66 (11.3%)	43 (8.3%)	31 (7.5%)	35 (8.9%)
Peapod	28 (1.5%)	11 (1.9%)	8 (1.5%)	3 (0.7%)	6 (1.5%)
Albertsons/Safeway	114 (6.0%)	31 (5.3%)	46 (8.9%)	22 (5.4%)	15 (3.8%)
FreshDirect	29 (1.5%)	16 (2.7%)	8 (1.5%)	5 (1.2%)	0 (0.0%)
Other	297 (15.6%)	51 (8.8%)	71 (13.7%)	70 (17.0%)	105 (26.7%)
Online grocery, delivery location					
Home	940 (49.3%)	246 (42.3%)	252 (48.6%)	219 (53.3%)	223 (56.7%)
Physical store location	568 (29.8%)	180 (30.9%)	164 (31.6%)	119 (29.0%)	105 (26.7%)
Both home and physical store location	397 (20.8%)	156 (26.8%)	103 (19.8%)	73 (17.8%)	65 (16.5%)
Online grocery, type of groceries ordered ^c^					
Fresh produce	357 (18.7%)	117 (20.1%)	62 (12.0%)	112 (27.2%)	66 (16.8%)
Canned produce	330 (17.3%)	107 (18.4%)	60 (11.6%)	101 (24.5%)	62 (15.8%)
Frozen produce	334 (17.5%)	104 (17.9%)	64 (12.4%)	101 (24.5%)	65 (16.5%)
Dairy products	331 (17.4%)	101 (17.4%)	65 (12.6%)	100 (24.2%)	65 (16.5%)
Soda or other sweetened drinks	326 (17.1%)	102 (17.5%)	63 (12.2%)	98 (23.8%)	63 (16.0%)
Bottled water	310 (16.3%)	107 (18.4%)	54 (10.4%)	98 (23.8%)	51 (13.0%)
Other beverages	208 (10.9%)	53 (9.1%)	41 (7.9%)	75 (18.2%)	39 (9.9%)
Bread, rice, or other types of grains	212 (11.1%)	50 (8.6%)	47 (9.1%)	76 (18.4%)	39 (9.9%)
Meat, poultry, or fish (fresh or frozen)	221 (11.6%)	47 (8.1%)	54 (10.4%)	69 (16.7%)	51 (13.0%)
Other frozen food	230 (12.1%)	49 (8.4%)	58 (11.2%)	66 (16.0%)	57 (14.5%)
Other canned food	319 (16.7%)	83 (14.3%)	72 (13.9%)	91 (22.1%)	73 (18.6%)
Desserts, snacks, or candy	335 (17.6%)	86 (14.8%)	76 (14.7%)	100 (24.2%)	73 (18.6%)
Other	142 (7.5%)	18 (3.1%)	41 (7.9%)	26 (6.3%)	57 (14.5%)
Online grocery, motivating factors ^c^					
Low prices	1083 (56.9%)	367 (63.1%)	313 (60.3%)	225 (54.7%)	178 (45.3%)
Variety of goods	743 (39.0%)	235 (40.4%)	190 (36.6%)	176 (42.8%)	142 (36.1%)
Good quality food	606 (31.8%)	210 (36.1%)	174 (33.5%)	119 (29.0%)	103 (26.2%)
Good produce selection	692 (36.3%)	179 (30.8%)	179 (34.5%)	168 (40.9%)	166 (42.2%)
Online convenience	28 (1.5%)	15 (2.6%)	9 (1.7%)	2 (0.5%)	2 (0.5%)
Having someone else select grocery items on my behalf	779 (40.9%)	224 (38.5%)	229 (44.1%)	189 (46.0%)	137 (34.9%)
Option for using SNAP benefits for online purchases	301 (15.8%)	108 (18.6%)	85 (16.4%)	56 (13.6%)	52 (13.2%)
Loyalty/frequent shopping program	661 (34.7%)	163 (28.0%)	177 (34.1%)	171 (41.6%)	150 (38.2%)
Inexpensive or no delivery fee	758 (39.8%)	202 (34.7%)	198 (38.2%)	192 (46.7%)	166 (42.2%)
Convenient pick-up or delivery options	103 (5.4%)	14 (2.4%)	16 (3.1%)	27 (6.6%)	46 (11.7%)
Other language options	190 (10.0%)	60 (10.3%)	54 (10.4%)	36 (8.8%)	40 (10.2%)
Other	800 (42.0%)	267 (45.9%)	220 (42.4%)	157 (38.2%)	156 (39.7%)
Online grocery, preventing or discouraging factors ^c^					
High prices	472 (29.1%)	94 (16.2%)	110 (21.2%)	145 (35.3%)	123 (31.3%)
Lack of variety of goods	166 (10.2%)	43 (7.4%)	37 (7.1%)	42 (10.2%)	44 (11.2%)
Poor quality food	148 (9.1%)	35 (6.0%)	35 (6.7%)	37 (9.0%)	41 (10.4%)
Poor produce selection	135 (8.3%)	23 (4.0%)	32 (6.2%)	39 (9.5%)	41 (10.4%)
Lack of social interaction	1040 (64.2%)	138 (23.7%)	202 (38.9%)	325 (79.1%)	375 (95.4%)
Not being able to touch and pick out the food itself	430 (26.5%)	79 (13.6%)	102 (19.7%)	130 (31.6%)	119 (30.3%)
No option for using SNAP benefits for online purchases	87 (5.4%)	16 (2.7%)	17 (3.3%)	31 (7.5%)	23 (5.9%)
No loyalty/frequent shopping program	455 (28.1%)	86 (14.8%)	122 (23.5%)	121 (29.4%)	126 (32.1%)
High delivery fees	252 (15.5%)	49 (8.4%)	68 (13.1%)	63 (15.3%)	72 (18.3%)
Not being home for delivery and/or deliveries being stolen	9 (0.6%)	2 (0.3%)	2 (0.4%)	4 (1.0%)	1 (0.3%)
Other language options	142 (8.8%)	24 (4.1%)	45 (8.7%)	30 (7.3%)	43 (10.9%)
Other	176 (10.9%)	36 (6.2%)	34 (6.6%)	50 (12.2%)	56 (14.2%)

Note: EBT = electronic benefit transfer; WIC = Special Supplemental Nutrition Program for Women, Infants, and Children; ^a^ Questions related to grocery shopping were derived from the United States Department of Agriculture National Household Food Acquisition and Purchase Survey or adapted from previous work by the authors. ^b^ Age group was calculated using quartiles. ^c^ Participants were allowed to check all choices that applied, so percentages exceed 100%.

**Table 3:** Model-based associations between sociodemographic characteristics and self-reported online grocery shopping (yes/no).

	OR (95% CI)
Age group, y	
18–20	**1.95 (1.52, 2.52)**
21–44	**1.50 (1.19, 1.90)**
45–64	1.05 (0.85, 1.29)
≥65	-
Female	0.92 (0.79, 1.07)
Household size, all	0.91 (0.82, 1.00)
Household size, children	**1.24 (1.07, 1.43)**
Hispanic, Latino, or Spanish	**1.63 (1.21, 2.19)**
Race	
White	-
Black	**1.52 (1.22, 1.89)**
American Indian or Alaska Native	0.63 (0.34, 1.18)
Asian, Native Hawaiian, or Pacific Islander	1.41 (0.79, 2.50)
Other	0.750.451.25
Education, high school or less	**0.83 (0.71, 0.97)**
Household annual income, <USD 20,000	**0.81 (0.68, 0.96)**
Marital status, married	**1.24 (1.02, 1.50)**
Employment status, employed	**1.43 (1.20, 1.69)**
SNAP, current participant	1.08 (0.91, 1.27)
Food insecure	**1.42 (1.20, 1.67)**
Responsible for most of food shopping	**1.32 (1.03, 1.70)**
Responsible for most of food preparation	**1.27 (1.01, 1.60)**
Fruit and vegetable intake, weekly (mean (SD))	
Q1 (3.7 (SD = 2.3))	**-**
Q2 (10.0 (SD = 1.6))	**1.35 (1.10, 1.65)**
Q3 (16.5 (SD = 2.3))	**1.54 (1.25, 1.90)**
Q4 (37.3 (SD = 21.6))	**1.97 (1.60, 2.43)**

Note: Boldface indicates statistical significance at *p* < 0.05. SNAP = Supplemental Nutrition Assistance Program.

**Table 4:** Model-based associations between age groups ^a^ and self-reported online grocery shopping behaviors and attitudes.

	18–33 Years (*n* = 857)	34–44 Years (*n* = 892)	45–59 Years (*n* = 889)	60+ Years (*n* = 888)
	OR (95% CI)	OR (95% CI)	OR (95% CI)	OR (95% CI)
Online grocery, ever in previous 12 months	**1.95 (1.52, 2.52)**	**1.50 (1.19, 1.90)**	1.05 (0.85, 1.29)	Referent
Online grocery, retailer ^b^				
Amazon	1.68 (1.22, 2.31)	1.16 (0.85, 1.59)	1.30 (0.97, 1.73)	Referent
Walmart	**1.86 (1.44, 2.41)**	**1.61 (1.26, 2.05)**	1.03 (0.82, 1.30)	Referent
Target	**4.26 (2.42, 7.47)**	1.96 (1.10, 3.48)	1.24 (0.68, 2.26)	Referent
Costco	1.76 (0.87, 3.54)	0.95 (0.46, 1.95)	1.07 (0.53, 2.16)	Referent
Kroger	1.73 (1.13, 2.65)	1.31 (0.87, 1.98)	1.07 (0.72, 1.61)	Referent
Whole Foods	2.55 (1.32, 4.95)	1.51 (0.78, 2.94)	0.87 (0.43, 1.76)	Referent
Aldi	1.41 (0.93, 2.14)	0.96 (0.63, 1.45)	0.71 (0.47, 1.07)	Referent
Publix	1.34 (0.77, 2.33)	0.97 (0.56, 1.68)	0.75 (0.44, 1.30)	Referent
Peapod ^c^	-	-	-	Referent
Albertsons/Safeway	1.99 (0.87, 4.51)	2.62 (1.22, 5.61)	1.68 (0.78, 3.63)	Referent
FreshDirect ^c^	-	-	-	Referent
Online grocery, delivery location				
Home	0.64 (0.45, 0.90)	0.88 (0.63, 1.22)	0.92 (0.67, 1.27)	Referent
Physical store location	1.22 (0.83, 1.77)	1.25 (0.87, 1.80)	1.08 (0.76, 1.53)	Referent
Both home and physical store location	1.53 (0.99, 2.36)	0.94 (0.61, 1.46)	1.05 (0.69, 1.60)	Referent
Online grocery, type of groceries ordered ^b^				
Fresh produce	1.68 (1.11, 2.54)	1.57 (1.06, 2.33)	0.87 (0.58, 1.30)	Referent
Canned produce	1.63 (1.06, 2.50)	1.52 (1.01, 2.27)	0.87 (0.58, 1.31)	Referent
Frozen produce	1.48 (0.97, 2.26)	1.43 (0.96, 2.12)	0.89 (0.59, 1.32)	Referent
Dairy products	1.45 (0.95, 2.21)	1.43 (0.96, 2.14)	0.90 (0.61, 1.35)	Referent
Soda or other sweetened drinks	1.59 (1.04, 2.44)	1.51 (1.01, 2.26)	0.94 (0.62, 1.40)	Referent
Bottled water	2.07 (1.33, 3.22)	1.78 (1.17, 2.72)	0.91 (0.59, 1.42)	Referent
Other beverages	1.14 (0.66, 1.95)	1.67 (1.03, 2.72)	1.00 (0.62, 1.64)	Referent
Bread, rice, or other types of grains	1.09 (0.64, 1.88)	1.71 (1.05, 2.78)	1.18 (0.74, 1.90)	Referent
Meat, poultry, or fish (fresh or frozen)	0.84 (0.49, 1.43)	1.26 (0.79, 2.01)	1.10 (0.71, 1.70)	Referent
Other frozen food	0.76 (0.45, 1.27)	0.97 (0.61, 1.54)	1.01 (0.66, 1.53)	Referent
Other canned food	1.10 (0.72, 1.69)	1.13 (0.75, 1.68)	0.98 (0.67, 1.44)	Referent
Desserts, snacks, or candy	0.99 (0.65, 1.51)	1.20 (0.82, 1.76)	0.94 (0.65, 1.36)	Referent
Online grocery, motivating factors ^b^				
Low prices	**2.34 (1.78, 3.08)**	**1.84 (1.42, 2.39)**	1.28 (1.00, 1.65)	Referent
Variety of goods	**1.73 (1.28, 2.34)**	1.37 (1.02, 1.83)	1.29 (0.98, 1.69)	Referent
Good quality food	**1.90 (1.36, 2.64)**	1.55 (1.13, 2.13)	1.12 (0.82, 1.53)	Referent
Good produce selection	1.06 (0.78, 1.43)	1.07 (0.81, 1.42)	1.02 (0.78, 1.32)	Referent
Online convenience ^c^	-	-	-	Referent
Having someone else select grocery items on my behalf	1.63 (1.19, 2.25)	**1.71 (1.27, 2.31)**	1.32 (0.99, 1.75)	Referent
Option for using SNAP benefits for online purchases	1.67 (1.07, 2.60)	1.42 (0.92, 2.19)	1.05 (0.68, 1.60)	Referent
Loyalty/frequent shopping program	0.90 (0.65, 1.23)	1.04 (0.77, 1.40)	1.09 (0.83, 1.43)	Referent
Inexpensive or no delivery fee	1.12 (0.83, 1.52)	1.13 (0.85, 1.49)	1.21 (0.93, 1.56)	Referent
Convenient pick-up or delivery options	0.51 (0.23, 1.15)	0.76 (0.39, 1.48)	0.83 (0.48, 1.42)	Referent
Other language options	1.27 (0.74, 2.18)	1.20 (0.72, 2.01)	0.80 (0.48, 1.34)	Referent
Online grocery, preventing or discouraging factors ^b^				
High prices	0.84 (0.59, 1.21)	0.88 (0.63, 1.23)	1.18 (0.88, 1.57)	Referent
Lack of variety of goods	1.06 (0.61, 1.84)	0.79 (0.46, 1.35)	0.85 (0.52, 1.37)	Referent
Poor quality food	0.61 (0.34, 1.11)	0.59 (0.34, 1.03)	0.76 (0.46, 1.26)	Referent
Poor produce selection	0.76 (0.39, 1.49)	0.76 (0.41, 1.40)	1.13 (0.68, 1.88)	Referent
Lack of social interaction	**0.34 (0.25, 0.45)**	**0.45 (0.35, 0.58)**	0.83 (0.67, 1.03)	Referent
Not being able to touch and pick out the food itself	0.76 (0.52, 1.12)	0.80 (0.56, 1.14)	1.06 (0.78, 1.43)	Referent
No option for using SNAP benefits for online purchases	1.02 (0.45, 2.34)	0.81 (0.36, 1.79)	1.49 (0.79, 2.82)	Referent
No loyalty/frequent shopping program	0.90 (0.62, 1.30)	1.07 (0.77, 1.49)	1.04 (0.77, 1.40)	Referent
High delivery fees	0.87 (0.55, 1.40)	1.04 (0.68, 1.59)	0.90 (0.60, 1.33)	Referent
Not being home for delivery and/or deliveries being stolen^c^	-	-	-	Referent
Other language options	0.85 (0.45, 1.60)	1.42 (0.84, 2.40)	0.71 (0.42, 1.22)	Referent

Note: Boldface indicates statistical significance at *p* < 0.0008. ^a^ Age group was calculated using quartiles. ^b^ Participants were allowed to check all choices that applied. ^c^ Non-estimable due to insufficient variation.

## Data Availability

The data presented in this study are available on request from the corresponding author.

## Supplementary Materials

The following supporting information can be downloaded at: https://www.mdpi.com/article/10.3390/nu14204427/s1, Supplemental File S1. Pre- and post-shopping task surveys.

## References

[1] United States Department of Agriculture (USDA) (2022). SNAP Data Tables. https://www.fns.usda.gov/pd/supplemental-nutrition-assistance-program-snap.

[2] Pasquali M. (2022). U.S. Online Grocery Retail. https://www.statista.com/topics/1915/us-consumers-online-grocery-shopping/#:~:text=In%20the%20United%20States%2C%20food,further%20in%20the%20upcoming%20years.

[3] Jilcott Pitts S.B., Ng S.W., Blitstein J.L., Gustafson A., Niculescu M. (2018). Online grocery shopping: Promise and pitfalls for healthier food and beverage purchases. Public Health Nutr..

[4] Rummo P.E., Bragg M.A., Yi S.S. (2020). Supporting Equitable Food Access During National Emergencies—The Promise of Online Grocery Shopping and Food Delivery Services. JAMA Health Forum.

[5] Chester J., Kopp K., Montgomery K.C. (2020). Does Buying Groceries Online Put SNAP Participants at Risk?—How to Protect Health, Privacy, and Equity.

[6] Dunn C.G., Bianchi C., Fleischhacker S., Bleich S.N. (2021). Nationwide Assessment of SNAP Online Purchasing Pilot State Communication Efforts During the COVID-19 Pandemic. J. Nutr. Educ. Behav..

[7] Food and Nutrition Service (FNS), United States Department of Agriculture (USDA) (2019). USDA Launches SNAP Online Purchasing Pilot. https://www.fns.usda.gov/pressrelease/2019/fns-000319.

[8] Jones J.W. (2021). COVID-19 Working Paper: Supplemental Nutrition Assistance Program and Pandemic Electronic Benefit Transfer Redemptions during the Coronavirus Pandemic.

[9] Brandt E.J., Silvestri D.M., Mande J.R., Holland M.L., Ross J.S. (2019). Availability of Grocery Delivery to Food Deserts in States Participating in the Online Purchase Pilot. JAMA Netw. Open..

[10] Foster I.S., Liu S.Y., Hoffs C.T., LeBoa C., Chen A.S., Rummo P.E. (2022). Disparities in SNAP online grocery delivery and implementation: Lessons learned from California during the 2020–21 COVID pandemic. Health Place.

[11] McGuirt J.T., Jilcott Pitts S.B., Labban J.D., Steeves B.A., Haynes-Maslow L., Henry S., Gustafson A. (2022). Evidence of Geospatial and Socioeconomic Disparities in Access to Online Grocery Shopping for Fresh and Frozen Produce in North Carolina. J. Acad. Nutr. Diet..

[12] Trude A.C.B., Lowery C.M., Ali S.H., Vedovato G.M. (2022). An equity-oriented systematic review of online grocery shopping among low-income populations: Implications for policy and research. Nutr. Rev..

[13] Trude A.C.B., Ali S.H., Lowery C.M., Vedovato G.M., Lloyd-Montgomery J.M., Hager E.R., Black M.M. (2022). A click too far from fresh foods: A mixed methods comparison of online and in-store grocery behaviors among low-income households. Appetite.

[14] Duffy E.W., Lo A.E., Hall M.G., Taillie L.S., Ng S.W. (2022). Prevalence and Demographic Correlates of Online Grocery Shopping: Results from a Nationally Representative Survey During the COVID-19 Pandemic. Public Health Nutr..

[15] U.S. Census Bureau Sex by Age, 2015–2019 American Community Survey 5-Year Estimates. https://data.census.gov/cedsci/table?d=ACS%205-Year%20Estimates%20Detailed%20Tables&tid=ACSDT5Y2019.B01001.

[16] (2022). Qualtrics. www.qualtrics.com/.

[17] Centers for Disease Control and Prevention (CDC), National Center for Health Statistics (NCHS) National Health and Nutrition Examination Survey Data. NHANES 2017–2018 Overview. https://wwwn.cdc.gov/nchs/nhanes/continuousnhanes/overview.aspx?BeginYear=2017.

[18] Economic Research Service (ERS), US Department of Agriculture (USDA) National Household Food Acquisition and Purchase Survey (FoodAPS). https://www.ers.usda.gov/foodaps.

[19] Martinez O., Tagliaferro B., Rodriguez N., Athens J., Abrams C., Elbel B. (2018). EBT Payment for Online Grocery Orders: A Mixed-Methods Study to Understand Its Uptake among SNAP Recipients and the Barriers to and Motivators for Its Use. J. Nutr. Educ. Behav..

[20] Rummo P.E., Naik R., Thorpe L.E., Yi S.S. (2021). Changes in diet and food shopping behaviors among Asian-American adults due to COVID-19. Obes. Sci. Pract..

[21] (2017). Behavioral Risk Factor Surveillance System (BRFSS) Fruit and Vegetable Module. https://www.cdc.gov/nutrition/data-statistics/data-users-guide.html.

[22] Holm S. (1979). A simple sequentially rejective multiple test procedure. Scand. J. Stat..

[23] Rogus S., Guthrie J.F., Niculescu M., Mancino L. (2020). Online Grocery Shopping Knowledge, Attitudes, and Behaviors Among SNAP Participants. J. Nutr. Educ. Behav..

[24] Berinsky A.J., Huber G.A., Lenz G.S. (2012). Evaluating online labor markets for experimental research: Amazon. Com’s Mechanical Turk. Political Anal..

[25] Jeong M., Zhang D., Morgan J.C., Ross J.C., Osman A., Boynton M.H., Mendel J.R., Brewer N.T. (2019). Similarities and Differences in Tobacco Control Research Findings From Convenience and Probability Samples. Ann. Behav. Med..

[26] Weinberg J.D., Freese J., McElhattan D. (2014). Comparing data characteristics and results of an online factorial survey between a population-based and a crowdsource-recruited sample. Sociol. Sci..

