# Supplementary material for: Age-Specific Differences in Online Grocery Shopping Behaviors and Attitudes among Adults with Low Income in the United States in 2021
Source: Nutrients. 2022 Oct 21;14(20):4427. doi: 10.3390/nu14204427 (PMC9609768; doi:10.3390/nu14204427)
Supplement: Supplementary file 1 [file nutrients-14-04427-s001.zip › nutrients-1958299-supplementary.pdf]

SUPPLEMENTAL FILE 1. Pre- and post-shopping task surveys

PRE-SHOPPING TRIP SURVEY

1. **How many people live in your household including yourself? Please do not include anyone who usually lives somewhere else.**[1] 1 person; 2 persons; 3 persons; 4 persons; 5 persons; More than 5 persons; Prefer not to answer

2. **How many children under the age of 18 live in your household?**[1] 0; 1; 2; 3; 4 or more

3. **Have you or anyone in your household ever received SNAP or Food Stamp benefits? This includes *any* SNAP benefits or Food Stamps, even if the amount was small and even if the benefits were received on behalf of children in the household.**[1] Yes; No; Don't know

4. **Do you or anyone in your household currently get SNAP benefits or Food Stamps? This includes *any* SNAP benefits or Food Stamps, even if the amount is small and even if the benefits are received on behalf of children in the household.**[1] Yes; No; Don't know

5. **What is your gender?**[1] Male; Female; Other
    a. **Please specify your gender if you chose 'Other' in the above question.**[1] ________

6. **What year were you born?** 1922-2003

7. **How old are you in years?**[1] ________

8. **Are you of Hispanic, Latino, or Spanish origin?** Yes; No; Prefer not to answer

9. **What race or races do you consider yourself to be? Please select one or more.**[1] American Indian or Alaska Native; Asian; Black or African American; Native Hawaiian or Pacific Islander; White; Other; Prefer not to answer
    a. **Please specify if you chose 'Other' in the above question.**[1] ________

10. **What is the highest grade or level of school you have completed or the highest degree you have received?**[1] Less than 9th grade; 9th to 12th grade - no diploma; High school graduate; GED or equivalent; Some college, no degree; Associate's degree; Bachelor's degree; Graduate or professional degree; Prefer not to answer

[1] Centers for Disease Control and Prevention (CDC). National Center for Health Statistics (NCHS). National Health and Nutrition Examination Survey Data. Hyattsville, MD: U.S. Department of Health and Human Services, Centers for Disease Control and Prevention, 2017-2018. https://wwwn.cdc.gov/nchs/nhanes/continuousnhanes/questionnaires.aspx?BeginYear=2017

11. **Now we are going to ask your total household income in 2020, including income from all sources, such as wages, salaries, Social Security or retirement benefits, help from relatives and so forth. Can you tell us that amount before taxes?**[1] Under $20,000; $20,000 to $39,999; $40,000 to $59,999; $60,000 to $79,999; $80,000 to $99,999; $100,000 to $119,999; $120,000 to $139,999; $140,000 to $159,999; $160,000 to $179,999; $180,000 to $199,999; $200,000 and over; Don't know; Prefer not to answer
    a. **You may not be able to give us an exact figure for your total household income, but can you tell us if this income in 2020 was**[1]**…** Less than $20,000; $20,000 or more; Don't know; Prefer not to answer

12. **Are you now married, widowed, divorced, separated, never married or living with a partner?**[1] Married; Widowed; Divorced; Separated; Never Married; Living with Partner; Prefer not to answer

13. **The next question is about your current job or business. Which of the following were you doing last week**[1]**…** Working at a job or business; With a job or business but not at work; Looking for work; Not working at a job or business; Part-time or full-time student; Prefer not to answer

14. **During the past month, how many times per <u>week</u> did you drink <u>100% PURE fruit juices</u>? Do not include fruit-flavored drinks with added sugar or fruit juice you made at home and added sugar to. Only include 100% juice.**[2]

    ____

15. **During the past month, not counting juice, how many times per <u>week</u> did you eat <u>fruit</u>? Count fresh, frozen, or canned fruit.**[2] ________

16. **During the past month, how many times per <u>week</u> did you eat <u>cooked or canned beans</u>, such as refried, baked, black, garbanzo beans, beans in soup, soybeans, edamame, tofu or lentils? Do NOT include long green beans.**[2] ________

17. **During the past month, how many times per <u>week</u> did you eat <u>dark green vegetables</u> for example broccoli or dark leafy greens including romaine, chard, collard greens or spinach?**[2] ________

18. **During the past month, how many times per <u>week</u> did you eat <u>orange-colored vegetables</u> such as sweet potatoes, pumpkin, winter squash, or carrots?**[2] ________

19. **Not counting what you just told me about, how many times per <u>week</u> did you eat <u>OTHER vegetables</u>? Examples of other vegetables include tomatoes, tomato juice or V-8 juice, corn, eggplant, peas, lettuce, cabbage,**

[2] Centers for Disease Control and Prevention (CDC). (2020, September 1). *Using the New Fruit and Vegetable Module in BRFSS.* Nutrition. https://www.cdc.gov/nutrition/data-statistics/using-the-new-BRFSS-modules.html

and white potatoes that are not fried such as baked or mashed potatoes.[2]

The next questions are about fruits and vegetables. Please think about all fruits and vegetables you ate during the past month (30 days), including meals and snacks. During the past month…

20. **How often did you drink 100% PURE fruit juices? Do not include fruit-flavored drinks with added sugar or fruit juice you made at home and added sugar to. Only include 100% juice.**[2] <1 time per week; 1 time per week; 2-3 times per week; 4-6 times per week; 1 time per day; 2 times per day; 3 or more times per day

21. **Not counting juice, how often did you eat fruit? Count fresh, frozen, or canned fruit.**[2] <1 time per week; 1 time per week; 2-3 times per week; 4-6 times per week; 1 time per day; 2 times per day; 3 or more times per day

22. **How often did you eat cooked or canned beans, such as refried, baked, black, garbanzo beans, beans in soup, soybeans, edamame, tofu or lentils? Do NOT include long green beans.**[2] <1 time per week; 1 time per week; 2-3 times per week; 4-6 times per week; 1 time per day; 2 times per day; 3 or more times per day

23. **How often did you eat dark green vegetables for example broccoli or dark leafy greens including romaine, chard, collard greens or spinach?**[2] <1 time per week; 1 time per week; 2-3 times per week; 4-6 times per week; 1 time per day; 2 times per day; 3 or more times per day

24. **How often did you eat orange-colored vegetables such as sweet potatoes, pumpkin, winter squash, or carrots?**[2] <1 time per week; 1 time per week; 2-3 times per week; 4-6 times per week; 1 time per day; 2 times per day; 3 or more times per day

25. **Not counting what you just told me about, how often did you eat OTHER vegetables? Examples of other vegetables include tomatoes, tomato juice or V-8 juice, corn, eggplant, peas, lettuce, cabbage, and white potatoes that are not fried such as baked or mashed potatoes.**[2] <1 time per week; 1 time per week; 2-3 times per week; 4-6 times per week; 1 time per day; 2 times per day; 3 or more times per day

26. **Out of the options below, which fruit do you purchase most often? (Pick one preferred option if you buy more than one option equally as often.)** Apples; Bananas; Grapes; Oranges; Strawberries

27. **Out of the options below, which <u>vegetable</u> do you purchase most often? (Pick one preferred option if you buy more than one option equally as often.)** Carrots; Lettuce; Onions; String Beans; Tomatoes

For each of the following statements, please tell me whether the statement was often true, sometimes true, or never true for your household in the last 12 months.[1]

28. **My household was worried whether our food would run out before we got money to buy more.[1]** True; Sometimes true; Never true; Don't know

29. **The food that we bought just didn't last, and we didn't have enough money to get more food.[1]** True; Sometimes true; Never true; Don't know

30. **Are you the person who does most of the <u>shopping for food</u> in your household?[3]** Yes; No; No one person is responsible

31. **Are you the person who does most of the <u>food preparation</u> in your household?[3]** Yes; No; No one person is responsible

32. **What kind of store do you buy most of your food from?[3]** Supermarket; Online-only retailer with delivery services like Amazon or FRESHDirect; Small or ethnic grocery store; Convenience store; Discount or big box store like Target or Walmart; Wholesale club like BJs, Costco, or Sam's Club; Dollar store like Dollar Tree or Dollar General; No one store type where I buy most of my food; Other
    a. **Please specify the store type if you chose 'Other' in the above question.[3]** ________

33. **What are your main reasons for shopping at the store (or stores) where you buy most of your food? Select all that apply.[3]** Low prices; Produce selection; Meat selection; Variety of foods; Close to home; Loyalty/frequent shopper program; Online convenience; Promotional offers; Other
    a. **Please specify if you chose 'Other' in the above question.[3]** ________

    34. **How often do you shop at that store in a typical month? Would you say[3]…** 1 times per month or less; 2-3 times per month; 1 time per week; More than 1 time per week

35. **In the last 12 months, how often did you shop for groceries online? This includes any groceries you ordered online from grocery stores, supermarkets, big box stores like Walmart, or online shopping websites like AmazonFresh or Instacart. It also includes groceries for curbside**

[3] USDA Economic Research Service. (2021). FoodAPS National Household Food Acquisition and Purchase Survey. Economic Research Service, Department of Agriculture. https://data.nal.usda.gov/dataset/foodaps-national-household-food-acquisition-and-purchase-survey.

pickup or delivery.[4] Never; 1 time per month or less; 2-3 times per month; 1 time per week; More than 1 time per week

36. **Where do you buy them in a typical month? Check all that apply.**[4] Amazon; Walmart; Target; Costco; Kroger; Whole Foods; Aldi; Publix; Peapod; Albertsons/Safeway; FreshDirect; Other
    a. **Please specify if you chose 'Other' in the above question.**[4]

    ____

37. **For groceries you order online, do you typically have them delivered to your home or do you pick them up in a physical store location?** Home; Physical store location; Both home and physical store location

38. **Please indicate what types of groceries you purchase online in a typical month. Check all that apply.**[4] Fresh produce; Canned produce; Frozen produce; Dairy products; Soda or other sweetened drinks; Bottled water; Other beverages; Bread, rice, or other types of grains; Meat, poultry, or fish (fresh or frozen); Other frozen food; Other canned food; Desserts, snacks, or candy; Other
    a. **Please specify if you chose 'Other' in the above question.**[4]

    ____

39. **What factors motivate you to buy groceries online in a typical month? Select as many as apply.**[4] Low prices; Variety of goods; Good quality food; Good produce selection; Online convenience; Having someone else select grocery items on my behalf; Option for using SNAP benefits for online purchases; Loyalty/frequent shopping program; Inexpensive or no delivery fee; Convenient pick-up or delivery options; Other language options; Other
    a. **Please specify if you chose 'Other' in the above question.**[4]

    ____

40. **What factors prevent or discourage you from buying groceries online? Select as many as apply.**[4] High prices; Lack of variety of goods; Poor quality food; Poor produce selection; Lack of social interaction; Not being able to touch and pick out the food itself; No option for using SNAP benefits for online purchases; No loyalty/frequent shopping program; High delivery fees; Not being home for delivery and/or deliveries being stolen; Other language options; Other
    a. **Please specify if you chose 'Other' in the above question.**[4]

    ____

---

[4] Martinez, O., Tagliaferro, B., Rodriguez, N., Athens, J., Abrams, C., & Elbel, B. (2018). EBT Payment for Online Grocery Orders: a Mixed-Methods Study to Understand Its Uptake among SNAP Recipients and the Barriers to and Motivators for Its Use. *Journal of nutrition education and behavior*, *50*(4), 396–402.e1. https://doi.org/10.1016/j.jneb.2017.10.003; Rummo, P. E., Naik, R., Thorpe, L. E., & Yi, S. S. (2021). Changes in diet and food shopping behaviors among Asian-American adults due to COVID-19. *Obesity science & practice*, *7*(3), 307–320. Advance online publication. https://doi.org/10.1002/osp4.485

41. **Thinking about all places your household shops for groceries, in a typical week when you do your largest shopping trip, about how much does your household spend on groceries? Your best guess is fine.**[4] less than $25; $25-$49.99; $50-$74.99; $75-$99.99; $100-$124.99; $125-$149.99; $150-$175.99; $175-$199.99; $200 and over

42. **How do you usually pay for groceries? (Please choose all options that apply).**[4] Cash; Credit/debit card; EBT card; WIC benefits

<u>POST-SHOPPING TRIP SURVEY</u>

Thank you for your order! You're almost done!

Please answer the following 8 questions about your experience shopping in the online grocery store then click submit and you will be 100% finished.

1. **Overall, how difficult or easy was it to use the online grocery store?** Very difficult; Difficult; Neither difficult nor easy; Easy; Very easy

2. **I could easily find all of the food and beverages I was looking for in the online grocery store.**[5] Strongly disagree; Somewhat disagree; Neither agree nor disagree; Somewhat agree; Strongly agree

3. **There were enough food and beverage options in the online grocery store.**[5] Strongly disagree; Somewhat disagree; Neither agree nor disagree; Somewhat agree; Strongly agree

4. **I was able to imagine doing my real-life shopping in the online grocery store.**[5] Strongly disagree; Somewhat disagree; Neither agree nor disagree; Somewhat agree; Strongly agree

5. **The food and beverages I purchased in this store were similar to my regular food and beverage purchases.**[5] Strongly disagree; Somewhat disagree; Neither agree nor disagree; Somewhat agree; Strongly agree

6. **This online supermarket felt like a real online grocery store.**[5] Strongly disagree; Somewhat disagree; Neither agree nor disagree; Somewhat agree; Strongly agree

7. **To what degree do you support having a grocer use your own preferences and past purchases to have healthy items, such as fruits and vegetables, appear in your cart when you log in, with the option to delete them?**

---

[5] Hall, M. G., Higgins, I., Grummon, A. H., Lazard, A. J., Prestemon, C. E., Sheldon, J. M., & Taillie, L. S. (2021). Using a Naturalistic Store Laboratory for Clinical Trials of Point-of-Sale Nutrition Policies and Interventions: A Feasibility and Validation Study. *International journal of environmental research and public health*, *18*(16), 8764. https://doi.org/10.3390/ijerph18168764

Strongly Oppose; Oppose; Somewhat Oppose; Neither Oppose nor Support; Somewhat Support; Strongly Support

8. **To what degree do you support having a grocer use your own preferences and past purchases to have healthy items, such as fruits and vegetables, appear in your cart when you log in, with the option to delete them and those items are discounted?** Strongly Oppose; Oppose; Somewhat Oppose; Neither Oppose nor Support; Somewhat Support; Strongly Support
